# Identification and expression profiling of microRNAs involved in the stigma exsertion under high-temperature stress in tomato

**DOI:** 10.1186/s12864-017-4238-9

**Published:** 2017-11-02

**Authors:** Changtian Pan, Lei Ye, Yi Zheng, Yan Wang, Dandan Yang, Xue Liu, Lifei Chen, Youwei Zhang, Zhangjun Fei, Gang Lu

**Affiliations:** 1 Key Laboratory of Horticultural Plant Growth, Development and Biotechnology, Agricultural Ministry of China, Department of Horticulture, Zhejiang University, Hangzhou, 310085 China; 2 Boyce Thompson Institute, Cornell University, Ithaca, NY 14853 USA; 3 USDA Robert W. Holley Center for Agriculture and Health, Ithaca, NY 14853 USA; 4 Zhejiang Provincial Key Laboratory of Horticultural Plant Integrative Biology, Zhejiang University, Hangzhou, 310085 China

**Keywords:** Tomato, Heat stress, MicroRNA, Stamen, Pistil, High-throughput sequencing

## Abstract

**Background:**

Autogamy in cultivated tomato varieties is a derived trait from wild type tomato plants, which are mostly allogamous. However, environmental stresses can cause morphological defects in tomato flowers and hinder autogamy. Under elevated temperatures, tomato plants usually exhibit the phenotype of stigma exsertion, with severely hindered self-pollination and fruit setting, whereas the inherent mechanism of stigma exsertion have been hitherto unknown. Numerous small RNAs (sRNAs) have been shown to play significant roles in plant development and stress responses, however, none of them have been studied with respect to stamen and pistil development under high-temperature conditions. We investigated the associations between stigma exsertion and small RNAs using high-throughput sequencing technology and molecular biology approaches.

**Results:**

Sixteen sRNA libraries of Micro-Tom were constructed from plants stamen and pistil samples and sequenced after 2 d and 12 d of exposure to heat stress, respectively, from which a total of 110 known and 84 novel miRNAs were identified. Under heat stress conditions, 34 known and 35 novel miRNAs were differentially expressed in stamens, and 20 known and 10 novel miRNAs were differentially expressed in pistils. GO and KEGG pathway analysis showed that the predicted target genes of differentially expressed miRNAs were significantly enriched in metabolic pathways in both stamen and pistil libraries. Potential miRNA-target cleavage cascades that correlated with the regulation of stigma exsertion under heat stress conditions were found and validated through qRT-PCR and RLM-5′ RACE.

**Conclusion:**

Overall, a global spectrum of known and novel miRNAs involved in tomato stigma exsertion and induced by high temperatures were identified using high-throughput sequencing and molecular biology approaches, laying a foundation for revealing the miRNA-mediated regulatory network involved in the development of tomato stamens and pistils under high-temperature conditions.

**Electronic supplementary material:**

The online version of this article (10.1186/s12864-017-4238-9) contains supplementary material, which is available to authorized users.

## Background

In flowering plants, the evolution from allogamy to autogamy is the most common feature in evolutionary history [1]. Autogamy is usually associated with the position of the anther relative to the stigma. For cross-pollination, the stigmatic surface of the pistil is exserted beyond its own anther and receives pollen from neighboring flowers, whereas self-pollinated flowers are typically characterized by a stigmatic surface that is recessed within its own anther [2]. Most wild tomato species exhibit allogamy and bear flowers with exserted stigmas, whereas cultivated tomato plants are autogamous with flush or inserted stigmas that help to complete self-fertilization [3]. Five tightly linked genes, including one controlling style length (*style2.1*), three involved in stamen length (*stamen2.1*, *stamen2.2*, and *stamen2.3*), and one affecting stamen architecture (*dehiscence2.1*), have been identified at the *se2.1* quantitative trait locus (QTL) through high-resolution mapping [4]. Among these five loci, *style2.1* is the major QTL accounting for the dominant variation in stigma exsertion, which occurs via the regulation of cell elongation of the pistil under normal conditions, whereas the functions of *stamen2.1*, *stamen2.2*, and *stamen2.3* remain unknown [2].

Multiple environmental stresses can cause severe defects in floral morphology and change the position of the anther relative to the stigma [5]. For tomato plants, during the reproductive development period, floral organs are more sensitive to environmental stresses than vegetative organs [6]. Grown at a relatively elevated temperature, tomato plants usually exhibit a high stigma exsertion rate, which strongly hinders self-pollination (autogamy), and further results in the failure of fruit set [7–9]. Previous observations have demonstrated that stigma exsertion in different tomato genotypes ranges from 25 to 55% under high-temperature conditions, indicating that this trait is genetically determined with incomplete dominance [10]. Furthermore, heat tolerance of tomato plants shows a robust negative correlation with the frequency of stigma exsertion under high-temperature conditions [10]. Sato et al. [11] suggested that it is the shortening anther instead of the elongating stigma that contributes to tomato stigma exsertion in response to high-temperature stress. So the molecular bases of stigma exsertion at elevated temperatures remain poorly understood.

MicroRNAs (miRNAs) are a class of single-stranded noncoding RNAs approximately 21 nucleotides (nt) in length that are involved in post-transcriptional gene silencing through the degradation of mRNAs or repression mRNA translation [12]. Since the first miRNA was identified in *Caenorhabditis elegans*, more than 28,000 miRNAs from 223 species have been deposited in the miRBase [13]. In plants, substantial evidence has demonstrated that miRNAs perform a critical role in multiple growth and development processes, including root, shoot, leaf, and flower development; phase switches; senescence; and biotic and abiotic defense responses [14–18]. For instance, miR395, miR399 and miR398 are induced under sulfate-, phosphate-, and copper-deprived conditions, respectively [19–21]. *Arabidopsis* miR172 targets *APETALA2-*like genes and further regulates flowering time and floral organ identity [22]. MicroRNA156b plays a critical role in the control of flower and fruit morphology in tomato through the regulation of meristem maintenance and the initial stage of fruit development [23]. In tomato, overexpression of *Arabidopsis* miR167a causes dramatic reproductive dysfunction in floral development and female fertility via the downregulation of *auxin response factors 6* (*ARF6*) and *ARF8* [24]. Some miRNAs (e.g., miR156, miR164, miR168, miR171, miR393, miR396, and miR398) are associated with a broad range of plant defense responses to stresses including drought, salt, and cold stresses [18]. More recently, based on high-throughput sequencing technology, abundant conserved and novel miRNAs that are responsive to heat stress were identified. In *Populus tomentosa*, 52 miRNAs were responsive to heat shock at 37 °C for 8 h, of which 41 were downregulated [25]. In rice, a total of 47 miRNAs were differentially expressed under heat-stress conditions [26]. A total of 36 miRNAs were identified in response to heat treatment (40 °C) for 2 h including miR172, miR156, and miR159 families in wheat [27, 28]. In *Brassica rapa,* five conserved miRNAs and four novel miRNA were responsive to heat stress (exposed to 46 °C for 1 h) [29]. These findings demonstrate that miRNAs are vital participants in the regulation of heat responses in plants. However, the miRNAs involved in this process in tomato plants remain poorly understood. Only four differentially expressed novel miRNAs have been identified from the pollen of tomato plants upon 38 °C treatment for 1 h [30]. No study has been performed to identify the heat-responsive miRNAs in stamens and pistils of tomato plants. Considering that stigma exsertion can result in severe reductions of the yield and quality of tomato plants as well as the huge potential for thermotolerant crop breeding, it is urgent and crucial to elucidate the intrinsic molecular mechanisms of stigma exsertion under heat-stress conditions.

In this study, high-throughput sequencing technology was employed to screen for potential miRNAs participating in stigma exsertion under heat stress, using samples derived from flower buds at the bicellular pollen stage in tomato plants. Bioinformatics combined with molecular biology approaches were utilized to investigate the expression patterns and putative functions of miRNAs derived from stamen and pistil libraries under 35 °C treatment, and we attempted to elucidate the potential miRNA-mediated regulatory mechanism of stigma exsertion under heat-stress conditions in tomato. Our study provides new insight into the inherent mechanism of stigma exsertion under high-temperature conditions.

## Results

### Analysis of small RNAs generated from deep sequencing

**Fig. 1:**
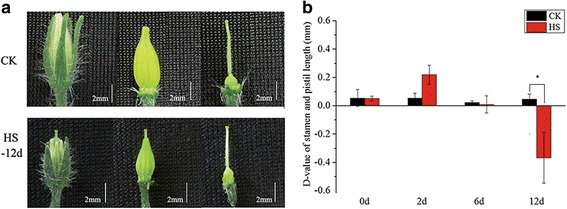
Characterization of tomato flower buds under heat stress conditions. CK: 25 °C treatment; HS: 35 °C treatment. **a** Phenotype of flower buds exposed to 35 °C for 12 d. **b** D-value of stamen and pistil length after exposure to 35 °C for 12 d. Values are mean ± SE of three independent replicates. Asterisks represent significant differences at *P* < 0.05

The flowers of tomato cultivar ‘Micro-Tom’ normally have recessed stigmas, and no stigma exsertion is observed under normal conditions (Fig. 1a). However, when exposed to high-temperature conditions, most of the flower buds at the bicellular pollen stage of this cultivar will show the phenotype of stigma exsertion at 10 days after heat treatment (Fig. 1a). To study the effects of high temperatures on the length of the style that protrudes from the anther cone, stigma exsertion levels defined as the D-value of stamen length and pistil length were evaluated at three different time points after high-temperature treatment. The D-value was close to zero for the first 6 days of high-temperature treatment. Notably, at 12 days after treatment, the value became negative with significant changes (*p*-value < 0.05), indicating that the stigma was exserted beyond its own stamen (Fig. 1b). The average length of stamens was 0.366 ± 0.180 mm shorter than that of pistil under the high-temperature condition.

To determine whether miRNAs were implicated in stigma exsertion under heat stress, 16 small RNA libraries were constructed for tomato stamens and pistils subjected to 25 °C and 35 °C as the control (CK) and heat-stress (HS) treatments, respectively, and then sampled at 2 d and 12 d after heat treatment with two biological replicates. Using Illumina sequencing technology, a total of 12.5 million, 11.3 million, 9.0 million, and 9.3 million raw reads were generated from CK-2d, HS-2d, CK-12d, and HS-12d libraries of tomato stamens, respectively (Table 1). For pistil libraries, a total of 11.4 million, 10.2 million, 13.8 million, and 11.0 million raw reads were obtained from CK-2d, HS-2d, CK-12d, and HS-12d libraries, respectively (Table 1). Biological replicates showed high correlations among sRNA expression profiles (Additional file 1: Table S1), indicating the high quality of the sRNA data. After removing the low-quality reads, such as poly(A) tails and short reads (<15 nt), 13.2 million (31.3%) and 19.5 million (42.1%) high-quality tags ranging from 15 to 40 nt in length were obtained from stamen and pistil libraries, respectively (Table1).

**Table 1:** Sequencing read statistics of the small RNA libraries of tomato stamens and pistils

Stamen	CK-2d		HS-2d		CK-12d		HS-12d	
	Stamen	Pistil	Stamen	Pistil	Stamen	Pistil	Stamen	Pistil
Raw reads	12,492,322^a^	11,367,006	11,307,154	10,153,953	9,025,192	13,838,872	9,285,582	10,969,105
Adapter & length filter	9,366,831	7,427,508	8,258,248	6,237,335	5,444,960	6,415,329	5,841,929	6,706,033
tRNA/snoRNA/snRNA	130,925	139,581	261,119	130,777	120,618	301,247	249,413	194,977
Clean reads	2,990,222	3,795,726	2,783,880	3,782,082	3,456,445	7,116,532	3,191,208	4,064,393
Unique reads	1,703,982	2,158,037	1,327,716	2,246,416	2,216,280	3,630,193	1500,192	1,974,354
Genome matched	1,138,085	1,469,847	832,665	1,573,237	1,603,960	2,515,561	1,046,428	1,370,401

^a^The data of reads represent average value of two biological replicates

**Fig. 2:**
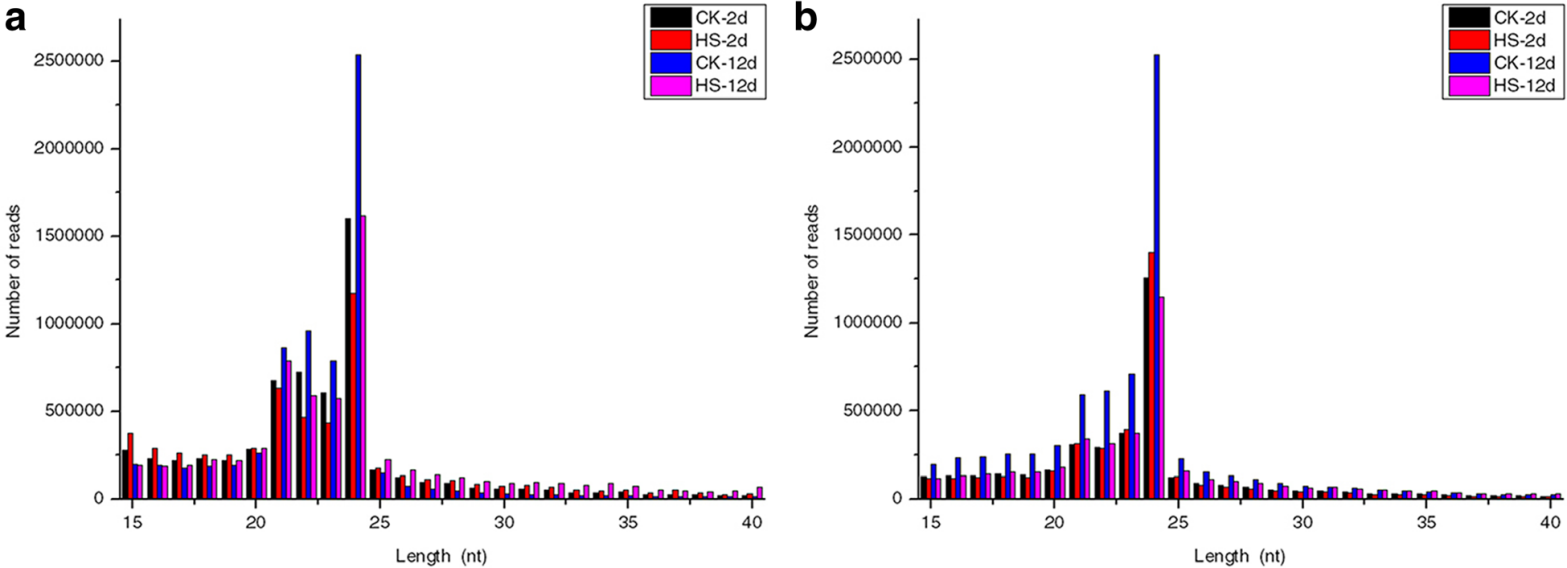
Length distribution of sRNAs in stamen and pistil libraries. **a** Length distribution of cleaned sRNA reads in each stamen library. **b** Length distribution of cleaned sRNA reads in each pistil library

The length distribution of cleaned sequence tags in each library is presented in Fig. 2. The clean sRNAs exhibited a similar pattern in the stamen and pistil libraries. Within the sRNA populations, the majority of total sRNA reads were 21–24 nt in length, accounting for over 60% of the total sequences. Furthermore, the 24 nt class sRNA were the most abundant, making up 27.9% and 33.8% of the stamen and pistil libraries, respectively, which is consistent with previous reports on *Arabidopsis* [31], rice [32], and tomato [33]. Notably, on average, the amounts of 21–24 nt sRNAs in stamen libraries was approximately 30% more than that in pistil libraries. However, the amount of 21–24 nt sRNAs decreased by 24.9% in the stamen library under heat-stress conditions for 2 d; in contrast, it increased by 7.6% in the pistil library (Fig. 2). Subsequently, continuous heat treatment for 12 d reduced 21–24 nt sRNAs by 30.7% and 50.9% in stamen and pistil libraries, respectively. The obvious changes in 21–24 nt sRNAs suggest that these sRNAs might be essential for heat-stress responses in tomato flower buds.

The base distribution ranging from the 1st to the 24th of all miRNA candidates was calculated (Additional file 2: Figure S1). Uridine, a characteristic of sRNAs recruited by AGO1, was mostly found at the 5′-terminal of miRNA candidates at an average prevalence of 50.4%. By contrast, only 8.9% of miRNA candidates had cytosine at the 5′-terminal, suggesting that these sRNAs are recruited by AGO5 [34].

### Identification of known and novel miRNAs

**Fig. 3:**
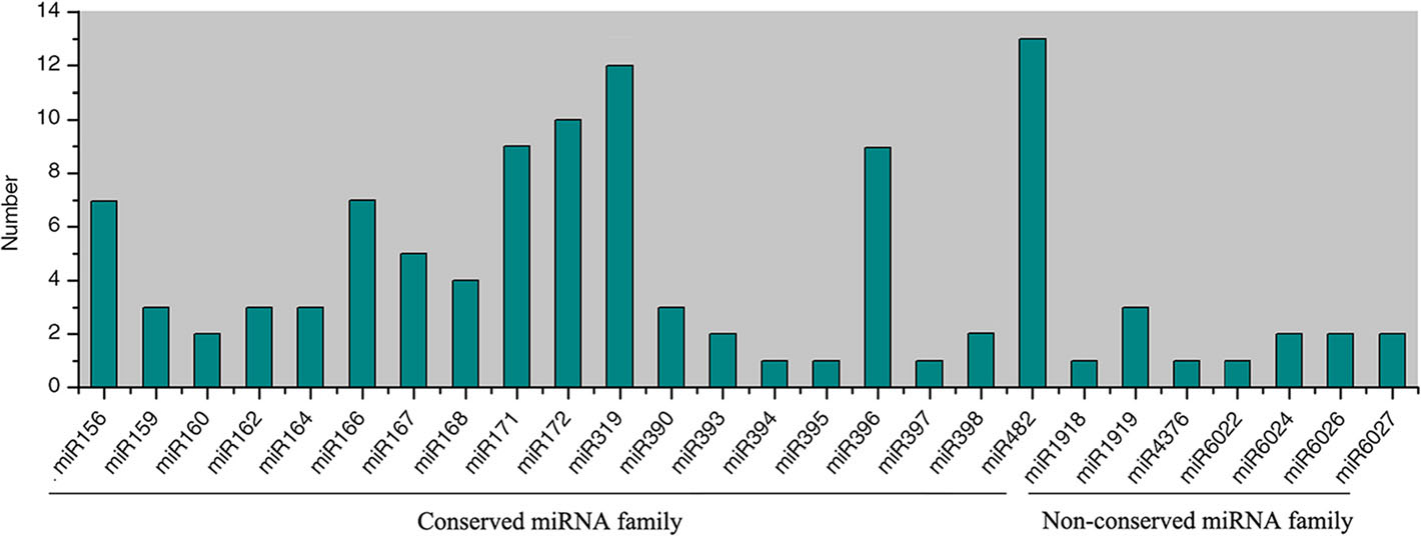
Number of identified known miRNAs in conserved and non-conserved miRNA families in tomato stamens and pistils

**Fig. 4:**
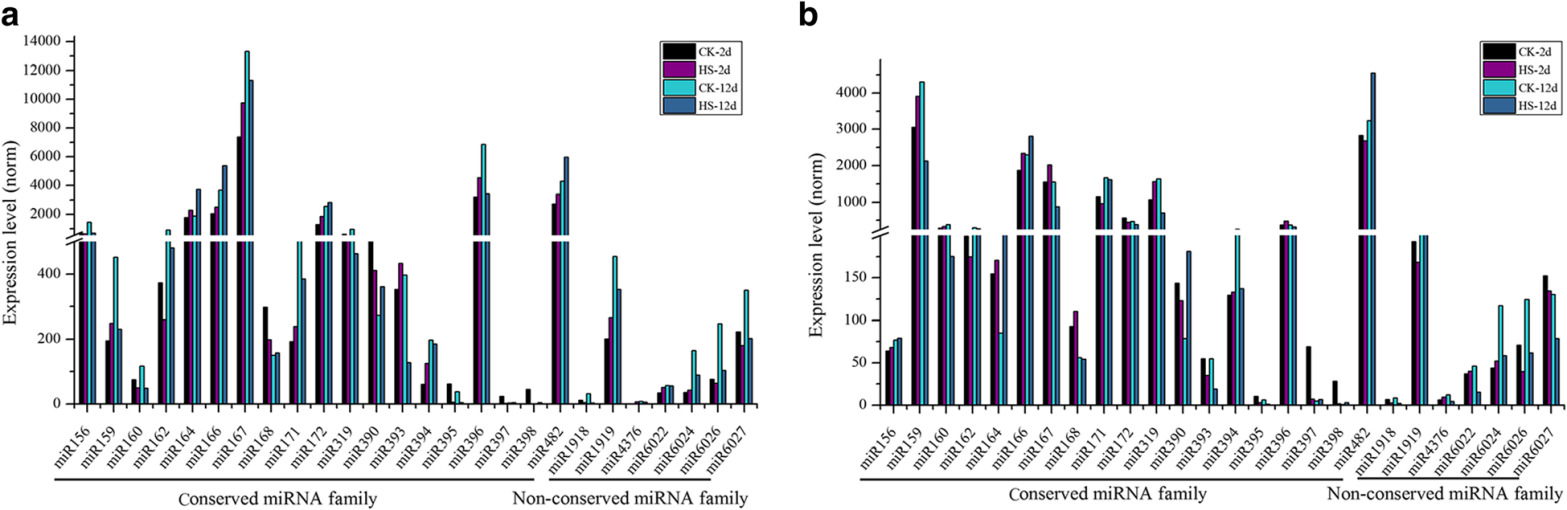
Abundance of known miRNA families in stamens (**a**) and pistils (**b**)

A total of 110 unique sequences belonging to 26 families in the stamen and pistil libraries were identified as known miRNAs previously deposited in the miRBase (version 21.0); of these, 19 miRNA families were highly conserved and the other seven were non-conserved (Fig. 3, Additional file 1: Table S2) [19]. The majority of the 26 miRNA families contained more than one member, and miR156, miR171, miR172, miR319, miR396, and miR482 had more than seven members. However, six miRNA families, namely, miR394, miR395, miR397, miR1918, miR4376 and miR6022, had only one member (Fig. 3). The numbers and compositions of conserved miRNA families were quite similar between stamen and pistil libraries. However, the expression levels of the miRNA families varied between stamen and pistil libraries (Fig. 4). For conserved miRNA families, the miR167, miR396, and miR482 families were predominantly expressed in the range of 2000 transcripts per million (TPM) clean tags to 14,000 TPM in the stamen libraries, whereas the miR159, miR166, and miR482 families were predominantly in the range of 1500 TPM to 4500 TPM in the pistil libraries. Within non-conserved families, the miR1919 family was the most abundant member in both stamen and pistil libraries. Under normal growth temperatures, analyses of the expression profiles of known miRNA members revealed that three miRNAs were observed exclusively in the stamen library (Additional file 1: Table S3). In addition, 28 known miRNAs were predominantly expressed in the stamen library (|log_2_ (HS/CK)| ≥ 1, *p*-value ≤0.05), whereas only two miRNAs, miR171a and miR159, were preferentially expressed in the pistil library (Additional file 1: Table S3).

A total of 84 novel miRNAs were identified (Additional file 1: Table S4). Among them, 39 miRNAs were divided into 14 groups owing to their high sequence similarity. The majority of these novel miRNAs exhibited a quite low expression level compared to known miRNAs, which is consistent with previous research on other plant species [31]. Among these novel miRNAs, ten and one miRNAs were only found in the stamen and pistil libraries, respectively (Additional file 1: Table S3). In addition, nine novel miRNAs were preferentially expressed in the stamen library (|log2 (HS/CK)| ≥ 1, *p*-value ≤0.05) (Additional file 1: Table S3).

The precursor lengths of 84 novel miRNAs ranged from 61 to 218 nt with an average length of 117 nt (Additional file 1: Table S4). MicroRNA sequences were generally located on each arm of the stem-loop hairpin structure. The identified pre-miRNAs had a minimum free energy (MFE) ranging from −16.23 kcal/mol to −145 kcal/mol with an average of −52.84 kcal/mol, which is consistent with previous reports indicating that a low MFE is characteristic of miRNA precursors [35].

### Identification of high temperature-responsive miRNAs in tomato stamen and pistil

**Fig. 5:**
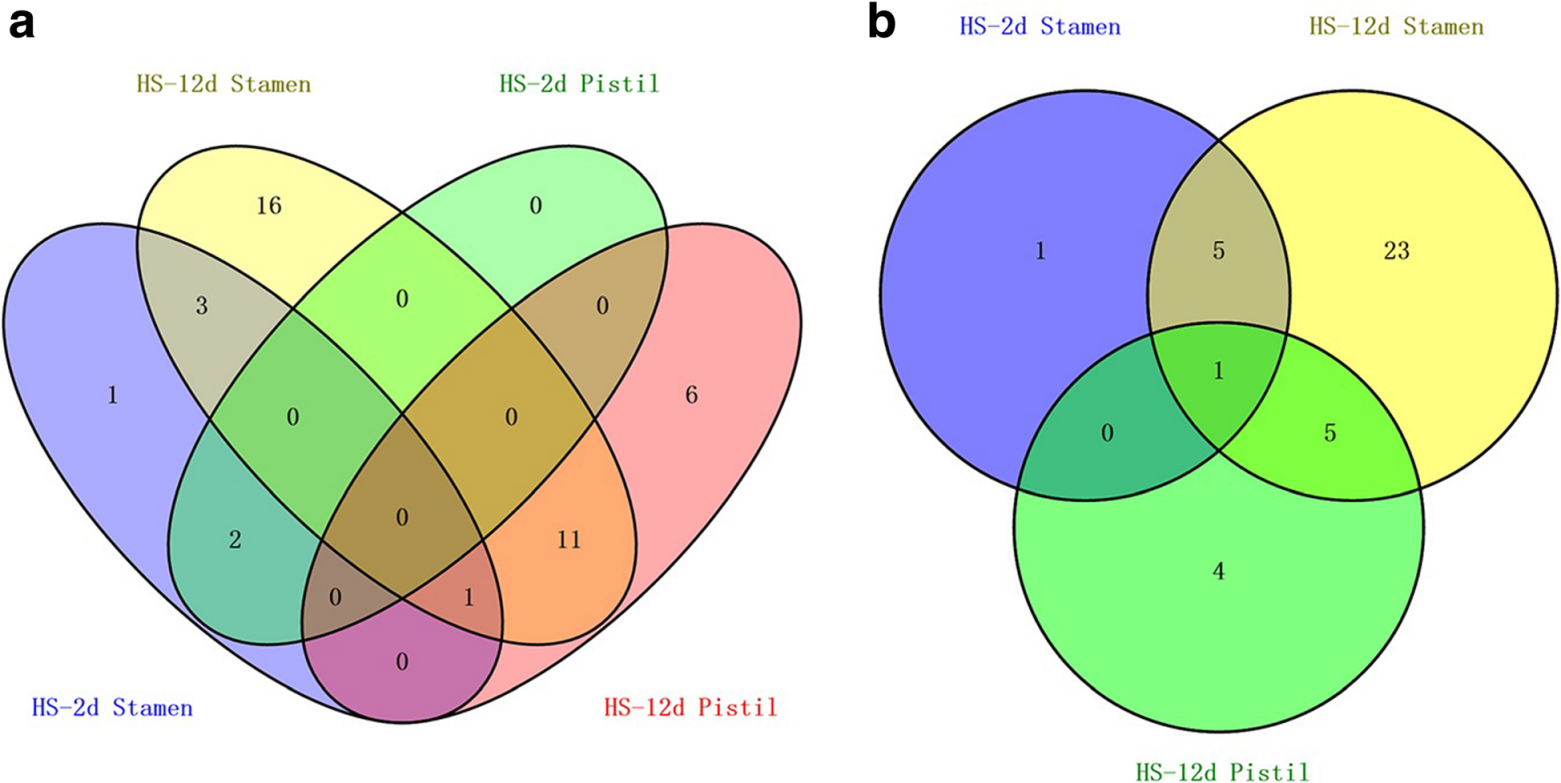
Venn diagram of differentially expressed conserved (**a**) and novel (**b**) miRNAs in stamens and pistils under heat stress conditions

A set of differentially expressed miRNAs were identified in the stamen and pistil libraries with the criteria of |log2 (HS/CK)| ≥ 1 and *p*-value ≤0.05. In the stamen library, a total of 34 unique known and 35 unique novel miRNAs showed differential expression, of which seven known (miR398a-5p, miR395a, miR398b-3p, miR397–5, pmiR160a-3, pmiR162a-5p and miR156e-3p) and seven novel miRNAs were significantly downregulated after 2 d of high-temperature treatment, whereas 31 known and 34 novel miRNAs were significantly down-regulated after 12 d of treatment (Additional file 1: Table S2 and Table S4). Among these differentially expressed miRNAs, four known (miR395a, miR160a-3p, miR162a-5p, and miR156e-3p) and six novel miRNAs were common in the HS-2d and HS-12d libraries (Fig. 5, Additional file 1: Table S2 and Table S4). In the pistil library, a total of 20 known and 10 novel miRNAs showed differential expression, of which two known miRNAs (miR398b-3p and miR397–5p) were significantly downregulated under heat stress for 2 d, and other miRNAs were detected at 12 d (Additional file 1: Table S2 and Table S4).

Notably, no miRNAs were significantly upregulated under heat stress in either the stamen or pistil libraries, indicating that long-term exposure to heat stress mainly suppressed the expression of miRNAs. Whereas two miRNAs, miR397–5p and miR398b-3p, were common in the stamen and pistil libraries at 2 d after heat-stress treatment, 12 known miRNAs belonging to the miR159, miR160, miR319, miR393, miR482, miR1918, miR6026, and miR6027 families (Fig. 5a, Additional file 1: Table S5) were common at 12 d after heat-stress treatment. This finding indicates that these miRNAs might play conserved functions in response to heat stress. By contrast, some miRNAs involved in the heat-stress response were specific to stamens or pistils. For instance, five (miR156e-3p, miR160a-3p, miR162a-5p, miR395a and miR398a-5p) and 19 (e.g., miR156b, miR156c, miR156e-3p, miR156e-5p, miR156d-5p, miR160a-3p, miR162a-5p) significantly expressed known miRNAs were specific to stamens in 2 d and 12 d libraries, respectively (Fig. 5a, Additional file 1: Table S6), whereas six significantly differentially expressed known miRNAs including miR172b, miR167a, miR319b, and miR482a were unique to pistils under heat-stress treatment for 12 d (Fig. 5a, Additional file 1: Table S7).

Considering that more miRNAs participated in the heat-stress response in tomato stamens than pistils, we concluded that tomato stamen may be more susceptible to high-temperature stress than the pistil. Furthermore, the significantly differentially expressed miRNAs between stamens and pistils may potentially be involved in the regulation of stamen and pistil development, which ultimately determines stigma exsertion under heat-stress conditions.

### Target prediction of differentially expressed miRNAs

To further evaluate the biological functions of miRNAs, the putative targets of differentially expressed known and novel miRNAs were identified (Additional file 1: Table S8 and Table S9). A total of 545 unique target genes of 38 known and 39 novel differentially expressed miRNAs were predicted using the psRNA Target Server [36]. Interestingly, each miRNA family had multiple predicted targets that were functionally divergent. Analysis of these predicted targets showed that approximately 10% of miRNA targets were annotated as transcription factors (TFs) playing crucial roles in the regulation of flower development, including *squamosa promoter-binding protein-like* genes (SPLs) [37], the APETALA 2 gene (AP2) [38], auxin response factors (ARFs) [39], and TCP TFs [40]. Some other targets were annotated as laccase genes and polygalacturonase genes, which are associated with plant cell wall construction [41, 42], as well as serine/threonine-protein kinases that likely participate in stress responses and signal transduction [43].

**Fig. 6:**
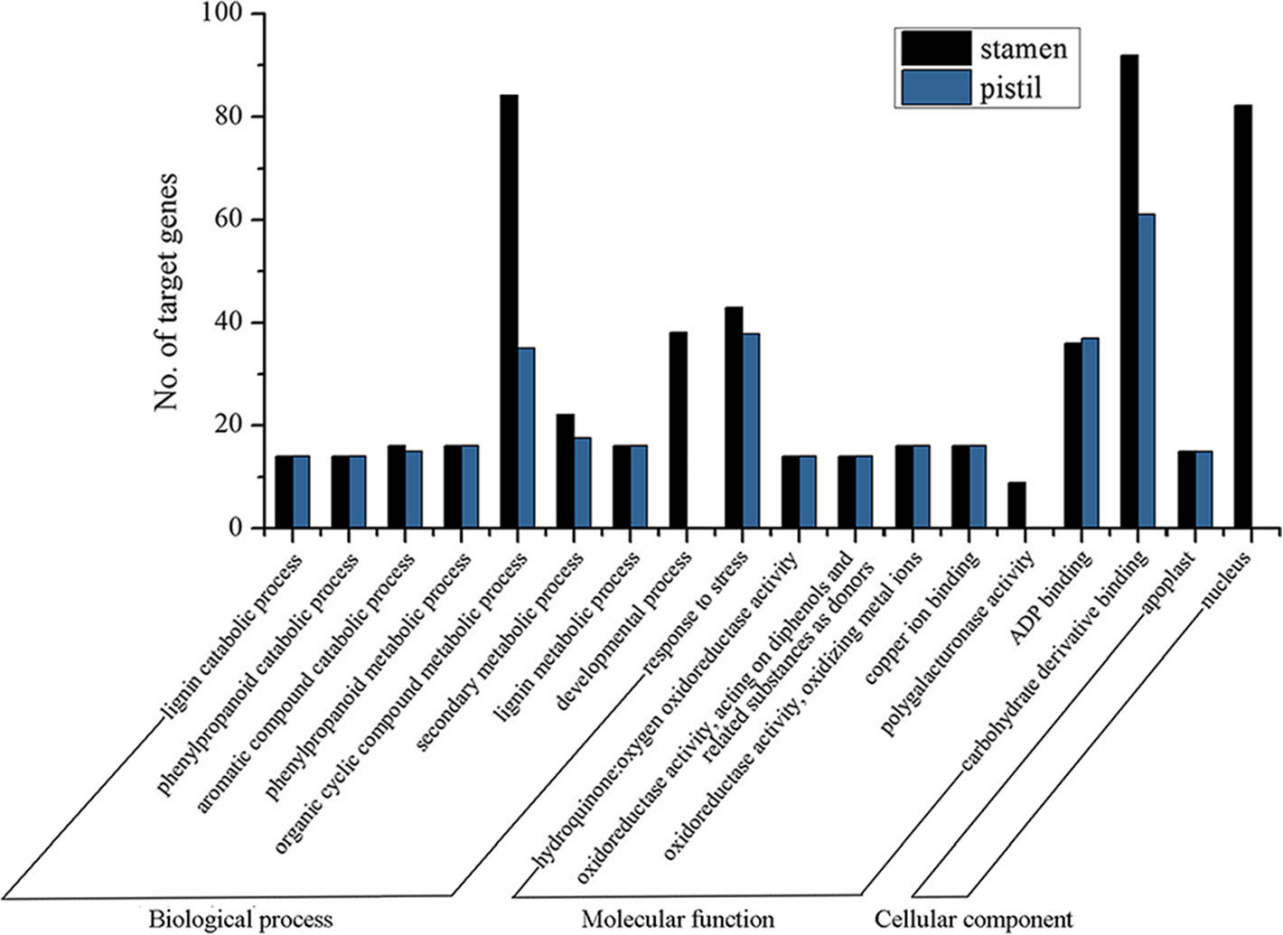
Gene Ontology annotation of putative targets of differentially expressed miRNAs in stamens and pistils

Gene Ontology (GO) analysis showed that these target genes of differentially expressed miRNAs were involved in distinct cellular and metabolic processes, of which the most over-represented GO terms are indicated in Fig. 6. The majority of GO terms were common to stamen and pistil libraries. Potential target genes of differentially expressed miRNAs were mainly involved in lignin metabolic processes (GO:0009808), responses to stress (GO:0006950), carbohydrate derivative binding (GO:0097367), and so on (Fig. 6). Meanwhile, these targets were mainly located in the apoplast and nucleus. However, developmental process (GO:0032502) and polygalacturonase activity (GO:0004650) were identified to be exclusively in stamen. In addition, KEGG pathway analysis was carried out for these target genes. The top 15 enriched pathways are shown in Table 2 with 70 target genes from stamens and 29 genes from pistils, of which metabolic pathways, biosynthesis of secondary metabolites, and plant hormone signal transduction were common to stamens and pistils. Intriguingly, purine metabolism and ribosome were enriched in stamens.

**Table 2:** Top-15 enriched KEGG pathways for target genes in tomato stamen and pistil libraries

Stamen		Pistil	
KEGG categories	No. of genes	KEGG categories	No. of genes
Metabolic pathways	23	Metabolic pathways	9
Biosynthesis of secondary metabolites	12	Biosynthesis of secondary metabolites	4
Purine metabolism	6	Zeatin biosynthesis	2
Ribosome	5	Phosphatidylinositol signaling system	2
Biosynthesis of amino acids	3	Inositol phosphate metabolism	2
Pyruvate metabolism	3	Fructose and mannose metabolism	1
Fatty acid metabolism	2	Cysteine and methionine metabolism	1
Glycolysis / Gluconeogenesis	2	Fatty acid metabolism	1
Fatty acid biosynthesis	2	Carbon metabolism	1
Pyrimidine metabolism	2	Plant hormone signal transduction	1
Cysteine and methionine metabolism	2	Sulfur metabolism	1
Glutathione metabolism	2	Ubiquitin mediated proteolysis	1
Folate biosynthesis	2	Peroxisome	1
Spliceosome	2	N-Glycan biosynthesis	1
Biosynthesis of amino acids	3	Inositol phosphate metabolism	2

### RLM-5′ RACE validation of miRNA and targets

**Fig. 7:**
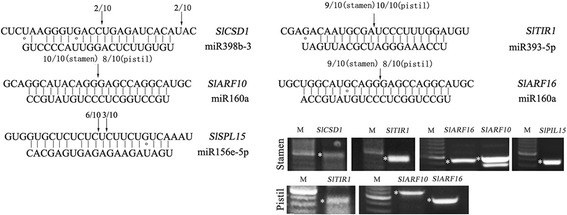
RLM-5′ RACE validation of miRNA target genes in stamens and pistils. Gene transcripts and miRNAs are in the 5′-3′ and 3′-5′ directions, respectively. Arrows denote the cleavage sites of target mRNAs, and numbers above them suggest the frequency (out of 10) of sequences observed at the exact miRNAs cleavage sites. M: marker; asterisk indicates PCR amplification products of decapped mRNA

The cleavage sites of interesting predicted target genes were validated in stamens and pistils using RLM-5′ RACE (Fig. 7). Four differentially expressed miRNAs, namely, miR398b-3, miR393-5p, miR160a, and miR156e-5p, were examined in stamens, and miR393-5p and miR160a were examined in pistils. The cleavage products of *SlARF10/16* and *SlTIR1*, predicted targets of miR160a and miR393-5p, respectively, were identified in both stamen and pistil libraries with the same cleavage sites (Fig. 7). In addition, the cleavage products of predicted targets *SlCSD1* and *SlSPL15* were identified in stamens. Sequence analysis showed that cleavage sites for most of the targeted transcripts were mapped to the paired miRNAs at the 10th or 11th nucleotide from the 5′-end, whereas *SlSPL15* could be simultaneously cleaved at the two sites by miR156e-5p.

### qRT-PCR analysis expression profiling of miRNAs and their targets

**Fig. 8:**
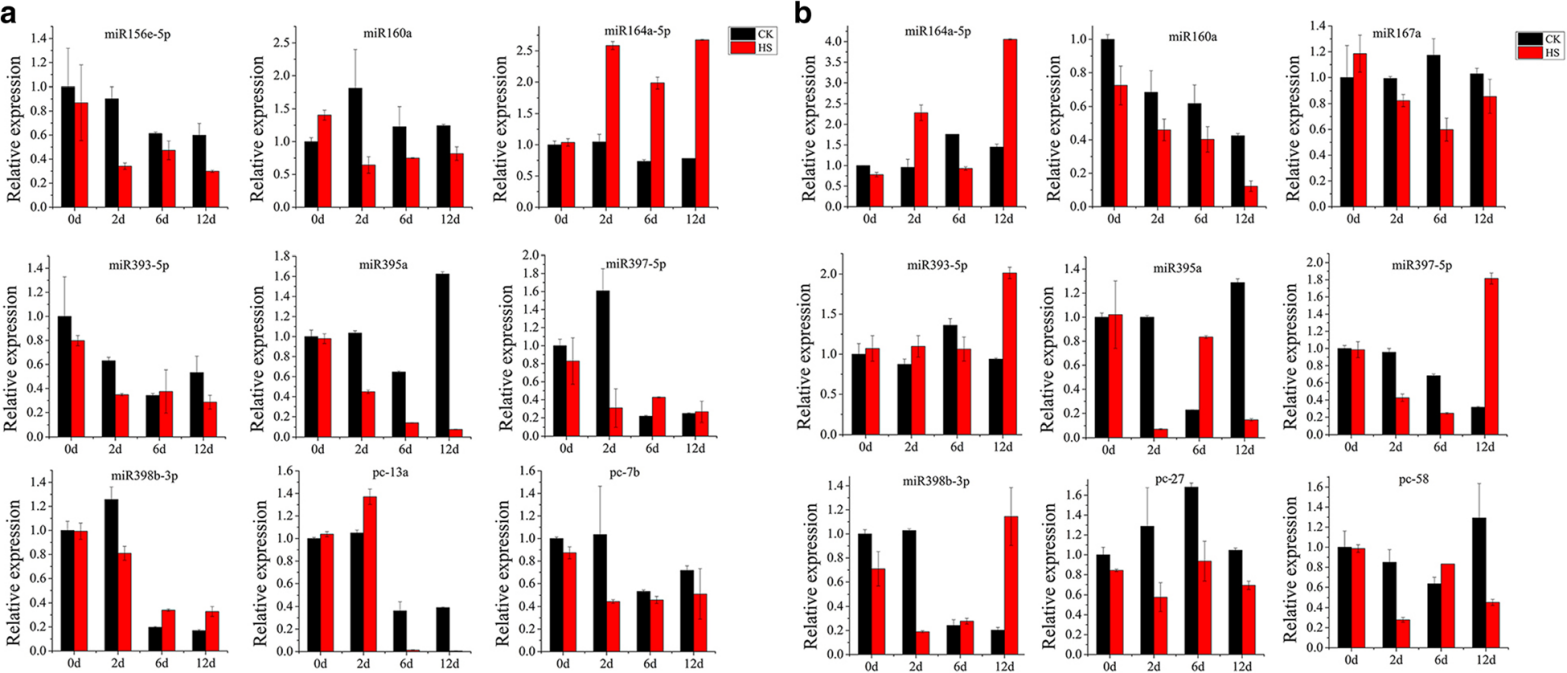
qRT-PCR validation of heat-responsive miRNAs in stamens (**a**) and pistils (**b**). *SnoU6* was used as the internal control. Each bar represents the mean ± SE of triplicated assays

The expression patterns of nine randomly selected miRNAs using qRT-PCR showed that most stamen miRNAs exhibited a decreasing trend after high-temperature treatment except for miR164a-5p, which was significantly increased at 2 d after high-temperature treatment (Fig. 8). The expression levels of miR156e-5p, miR160a, miR393-5p, miR397–5p, miR398b-3p, and pc-7b (pc: predicted candidates of novel miRNA) showed significant reduction at 2 d after high-temperature treatment, although these miRNAs exhibited no differential expression at the later stages. However, miR395a was continuously downregulated from 2 to 12 d of high-temperature treatment, whereas pc-13a was markedly decreased after 6 d of treatment. Similar to stamens, pistil miRNAs were generally downregulated in response to heat-stress treatment at 2 d after heat-stress treatment except for miR164a-5p, which was strongly upregulated at both 2 d and 12 d of treatment. In addition, miR395a, miR397–5p, miR398b-3p, and pc-27 were markedly downregulated at 2 d after heat-stress treatment, whereas miR397–5p and miR398b-3p were strongly increased at 12 d after heat-stress treatment. Overall, the qRT-PCR results were consistent with the sequencing data (Additional file 2: Figure S2).

**Fig. 9:**
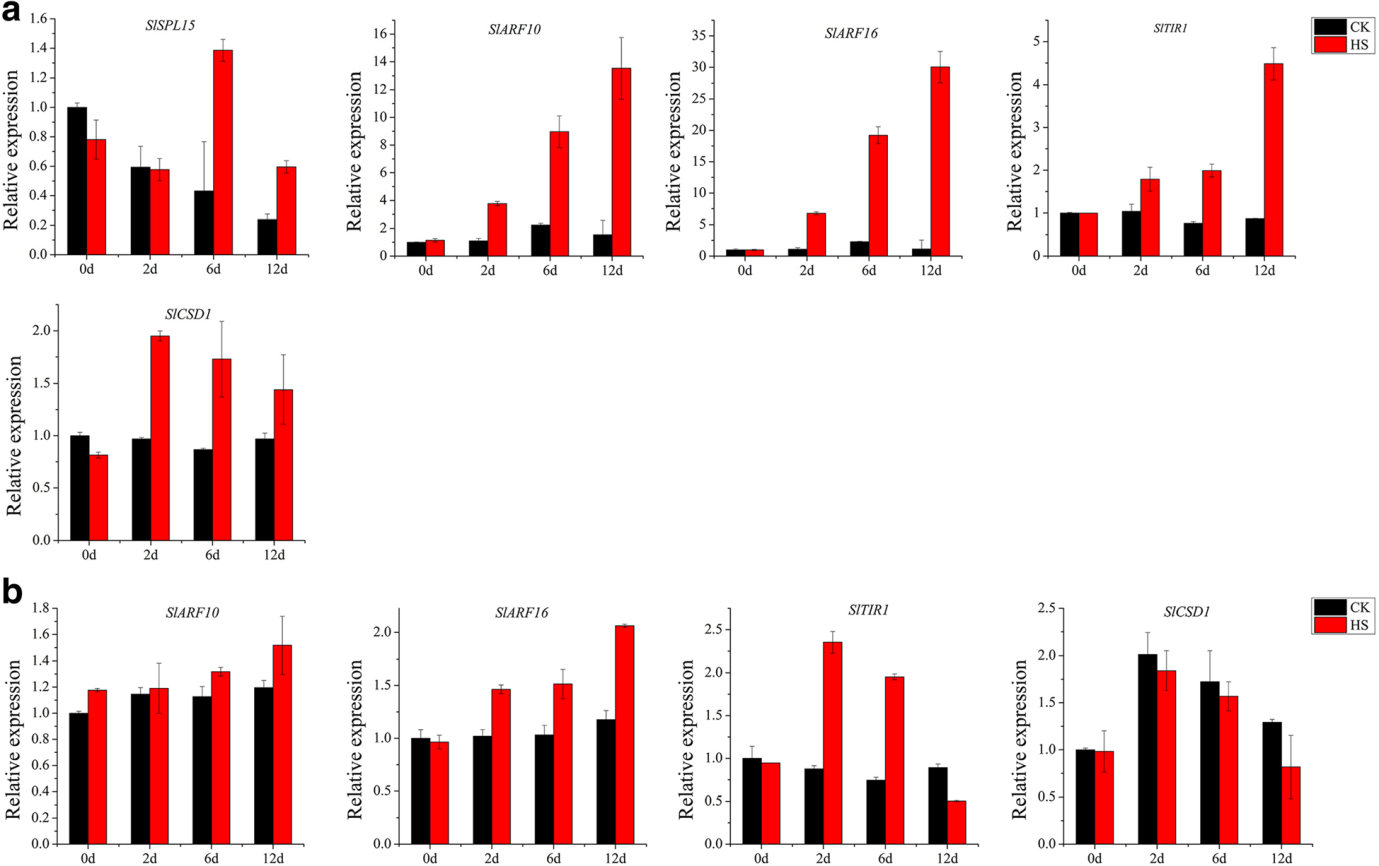
qRT-PCR analysis of miRNA target genes in stamens (**a**) and pistils (**b**). *SlUbi3* was used as the internal control. Each bar represents the mean ± SE of triplicated assays

In general, miRNAs and their target genes show contrasting expression patterns. Thus, the expression profiles of five target genes, *SlSPL15* for miR156, *SlARF10* and *SlARF16* for miR160, *SlTIR1* for miR393, and *SlCSD1* for miR398, were analyzed in stamens and pistils. The expression patterns of these five target genes showed significant increasing trends under high-temperature treatment, with a markedly negatively correlation with the expression patterns of their corresponding miRNAs, except for *SlCSD1* in pistils (Figs. 8 and 9). *SlCSD1* was up-regulated in stamen under heat-stress treatment, and there were no significant changes in pistils.

## Discussion

### High-throughput sequencing of small RNAs in stamens and pistils of tomato plants

In responding to environmental stresses, plants have evolved corresponding mating behaviors including physiological and morphological adaptations [5]. In tomato plants, the most striking change in floral morphology is the stigma exsertion, which strongly hinders self-pollination (autogamy) and substantially reduces the yield and quality [7–9]. MicroRNAs are important regulators implicated in plant growth and development processes and stress responses [17, 44]. In recent years, a large number of conserved and species-specific miRNAs have been identified in many important crops [45]. In tomato, a few miRNAs have been identified in the fruits and leaves [46, 47], and some miRNAs respond to fungi infection [48] and ABA treatment [49], however, the profiles of miRNAs in tomato flower organs in response to high temperature remain uninvestigated. To figure out whether miRNAs participate in the regulation of tomato stigma exsertion under high-temperature conditions, 16 sRNA libraries from stamens and pistils of tomato flower buds (at the bicellular pollen stage) were constructed to identify the heat-responsive miRNAs using high-throughput sequencing technology.

Tens of millions of sRNA reads were generated from each library, which is consistent with previous reports on tomato [50, 51]. The compositions of sRNAs are usually instrumental in elucidating the function and activity of different categories of sRNAs in specific tissues or species [52]. Among clean reads, the length distribution of sRNAs was mainly concentrated at 21–24 nt, and the 24 nt sRNAs were the most abundant class (Fig. 2). Similar results have been observed other studies on tomato [50] and in other plant species including *Arabidopsis* [53] and rice [45]. Remarkably, the 24 nt sRNAs were significantly altered under heat-stress treatment (Fig. 2). After the plants were exposed to elevated temperatures for 2 d, the 24 nt sRNAs were reduced by 26.6% in the stamen library but not in the pistil library. However, at 12 d after high temperature treatment, the reads of the 24 nt sRNA class decreased significantly both in the stamen and pistil libraries. These data suggest that the 24 nt sRNAs might be implicated in the response to high-temperature stress in tomato flower buds. As multi-functional molecules, transfer RNAs (tRNAs) play crucial roles in the control of multiple cellular metabolism processes, and their derived fragments participate in stress responses [54, 55]. In stamens, the abundance of sRNAs derived from tRNAs in the HS-2d and HS-12d libraries were nearly twice that of the corresponding control libraries (Additional file 2: Figure S3). By contrast, in pistils, the abundance of tRNA-derived sRNAs showed no significant change; it even decreased by 36.6% at 12 d after heat-stress treatment.

In this study, a total of 110 known miRNAs belonging to 26 families were identified, which included the most conserved miRNA families previously identified in tomato (Fig. 3) [33, 50, 56]. Furthermore, the conserved miRNA families were much more abundant and had more miRNA members than non-conserved miRNA families (Figs. 3 and 4). In the stamen library, the miRNA167 and miRNA396 families were the most abundant, whereas in the pistil library, the miRNA159 and miRNA482 families were the most abundant (Fig. 4). Interestingly, a total of 84 novel miRNAs were identified from stamen and pistil libraries with relatively low expression, which is consistent with previous reports that species-specific miRNAs are usually expressed at a lower level [53]. Among these novel miRNAs, ten were unique to the stamen library and only one unique miRNA was found in the pistil library (Additional file 1: Table S3). A total of 779 novel miRNAs had been identified in tomato in previous reports [30, 33, 48–50, 57–59], but among them, only 54 novel miRNAs were shared between at least two separate studies. The inconsistencies of predicted miRNAs among these studies are largely due to differences in the organs/tissues sampled and developmental stages. It is worth pointing out that all 84 novel miRNAs were tomato-specific (Additional file 1: Table S4), and 13 were consistent with previously studies on tomato [46–49].

### Heat-responsive miRNAs in stamen and pistil libraries

Characterization and comparative profiling of miRNAs provide the foundation for unraveling the regulatory networks of miRNA-mediated stigma exsertion under high temperatures. In this study, expression analysis showed that 69 miRNAs generated from stamen libraries significantly changed under high-temperature treatment, whereas only 30 miRNAs were found to be differentially expressed in pistil libraries (Additional file 1: Table S2 and Table S4). This discrepancy indicates that stamen may be more sensitive to high temperatures than pistil. It is worth noting that almost all differentially expressed miRNAs under heat stress were downregulated. Genome-wide identification of microRNAs involved in heat-stress responses has previously been conducted in *P. tomentosa* [25], *Triticum aestivum* [27], *B. rapa* [29], and tomato [30]. However, the conserved miRNAs exhibited a different response pattern in diverse plant species under high-temperature stress. For example, miR160, miR168, and miR169 increased under 40 °C for 1 h in leaves of wheat seedlings, whereas pto-miR160, pto-miR168, pto-miR169a-b, and pto-miR169n-t showed a significant reduction in *P. tomentosa* subjected to 37 °C for 8 h [25, 27]. These results suggested that the response model of miRNAs might be specific to tissues or species.

A deeper understanding of the functions of miRNAs would be facilitated by the identification of their corresponding targets. In the present study, a total of 482 and 265 unique target genes of differentially expressed miRNAs in stamen and pistil libraries under heat-stress treatment were predicted, respectively (Additional file 1: Table S8 and Table S9). GO analysis showed that the majority of GO terms were common to the stamen and pistil libraries (Fig. 6), indicating that similar responses to heat-stress treatment happened in both stamens and pistils, and the highly represented terms included ‘response to stress’ and ‘lignin catabolic process’. However, GO terms such as ‘developmental process’ and ‘polygalacturonase activity’ were unique to the stamen library. Consistently, KEGG analysis showed that ‘metabolic pathways’ and ‘biosynthesis of secondary metabolites’ were the most enriched pathways in both stamens and pistils, indicating that the target genes involved in the metabolism of flower buds are significantly regulated when exposed to high-temperature stress (Table 2).

### MicroRNAs possibly involved in the regulation of heat-induced stigma exsertion in tomato plants

**Fig. 10:**
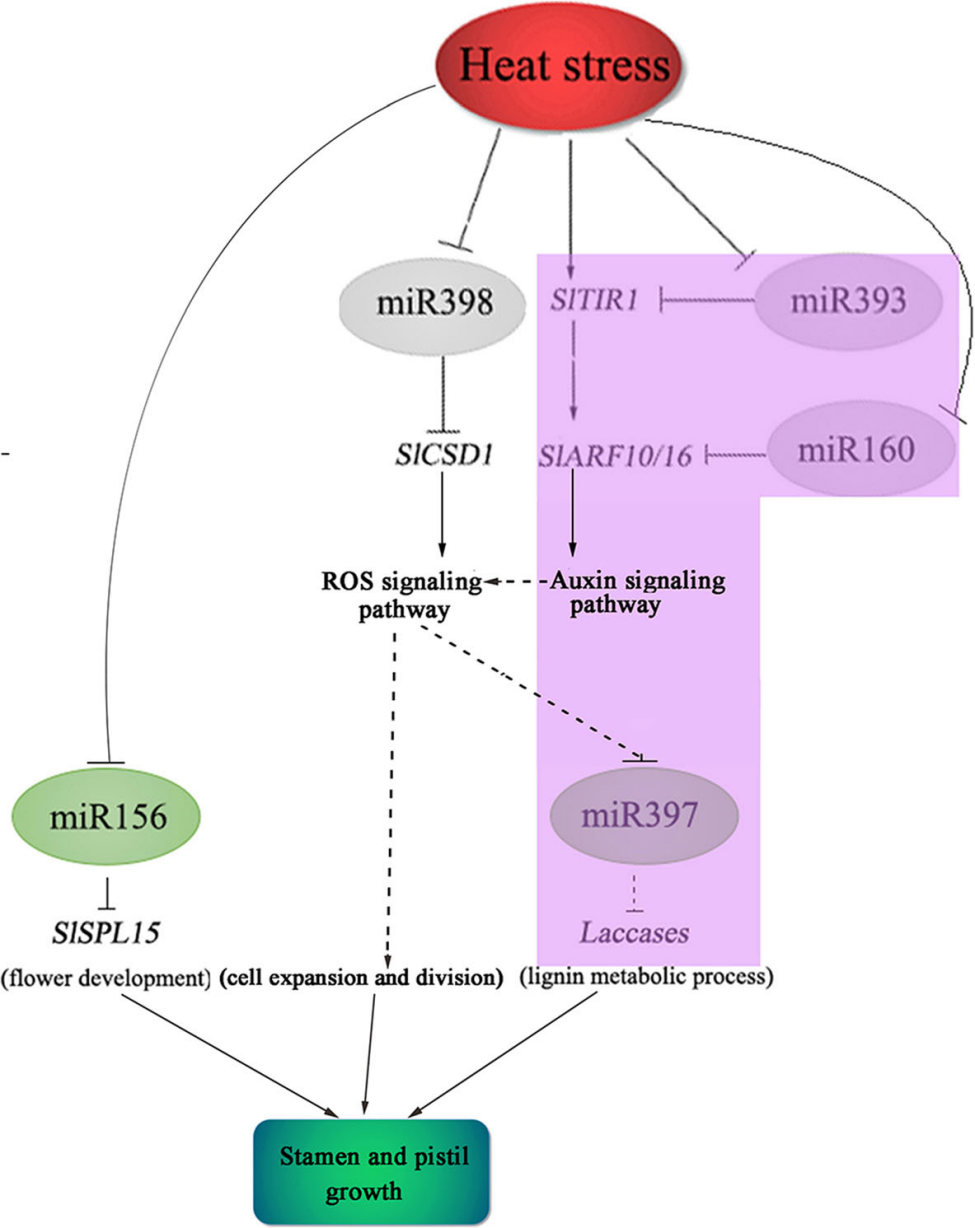
Schematic presentations of potential miRNA-target pairs implicated in the regulation of stigma exsertion under heat-stress condition. MiRNAs mediating responses in stamens are shown in the figure, and those in purple shading can also be observed in pistils. Dotted lines indicate the correlation between miRNAs and target genes remains to be further characterized

Analysis of all obtained differentially expressed miRNAs showed that 20 miRNAs were shared between the two libraries, whereas 49 and 10 miRNAs were unique to the stamen and pistil libraries, respectively (Additional file 1: Tables S5-S7). Among these miRNAs, miR398b-3p, miR393-5p, miR160a, miR156e-5p, and miR397–5p were of particular interest as their predicted targets play crucial roles in plant signal transduction, flower development, and cell wall architecture [21, 41, 60, 61]. RLM-5′ RACE validated that miR398b-3p, miR393-5p, miR160a, and miR156e-5p were active and directed the cleavage of their targets, except for miR397–5p (data not shown). A schematic presentation of these miRNA-target cleavage cascades and their roles in the regulation of heat responses and stigma exsertion is shown in Fig. 10.

Previous studies have demonstrated that miR398 targets two closely related Cu/Zn superoxide dismutases, CSD1 and CSD2, which are essential for promoting the production of superoxide dismutases (SODs) and further alleviating stress-induced damage in plants [21]. Under oxidative stress conditions, downregulated miR398 would increase the expression of *CSD1* and promote defense against the accumulation of reactive oxygen species (ROS) [62]. In our study, under heat-stress conditions, *SlCSD1* was negatively correlated with miR398b-3p levels in stamens (Figs. 8 and 9). RLM-5′ RACE detected two cleavage sites of *SlCSD1* mRNA; one was located at the complementary site of miR398b-3p and the other was adjoined to the complementary site (Fig. 7), indicating that the cleavage sites of miR398b-3p may be diverse, as reported in *Arabidopsis* [63]. These observations suggested that the miR398b-3p-*SlCSD1* cleavage cascade is tightly linked to the signal of high-temperature stress responses in tomato stamens. Meanwhile, miR393-5p and miR160a were found to target auxin receptor F-box protein *SlTIR1* and ARFs, respectively, which play critical roles in auxin-mediated signaling and plant development [60, 64]. When auxin binds to TIR1, it promotes the ubiquitination and degradation of auxin/indole-3-acetic acid (Aux/IAA) transcriptional repressors, thereby allowing ARFs to activate auxin-responsive genes [65, 66]. Previous studies have demonstrated that miR160 targets ARF10, ARF16, and ARF17 to modulate the expression of early auxin response genes [67, 68]. RLM-5′-RACE analysis in our study showed that miR393-5p directed the cleavage of *SlTIR1* transcripts*,* whereas miR160a guided the cleavage of *SlARF10* and *SlARF16* transcripts (Fig. 7). qRT-PCR and sequencing data showed that the levels of miR393-5p and miR160a in stamens and pistils were negatively correlated with the expression of *SlTIR1* and *SlARF10/16*, respectively (Figs. 8 and 9, Additional file 1: Table S2). These observations suggest that miR393-5p/*SlTIR1* and miR160a/*SlARF10/16* cleavage cascades mediated by auxin signaling were activated by heat-stress treatment.

A series of *SPL* TFs are targeted by the miR156 family, and the miR156/SPL module emerges as a pivotal regulator covering multiple aspects of plants, including the timing of phase changes, leaf development, organ size, fertility, and responses to stresses [69]. In *Arabidopsis*, overexpression of miR156b results in serious defects in juvenile-to-adult phase transitions and inflorescence architecture via the strong repression of *SPL9* and *SPL15* [70]. Our sequencing data showed that five miR156 family members were significantly downregulated under heat-stress treatment (Additional file 1: Table S2). qRT-PCR further proved that downregulated miR156e-5p was negatively correlated with an increase in its predicted target *SlSPL15*. These results imply that the miR156/SPL module might function in the regulation of stamen development under heat-stress conditions.

Laccases are involved in lignin polymerization, which is vital for the integrity of plant cell walls, and ptr-miR397a has been proven to be a negative regulator of laccase genes, thus affecting lignin content [41]. In *Arabidopsis*, overexpression of miR397b causes a reduction in lignin content through the downregulation of *LAC4* transcripts, thus changing cell size and cell mechanical properties [71, 72]. In our study, *SlLAC4* was also predicted to be cleaved by miR397–5p (Additional file 1: Table S8). qRT-PCR analysis showed that the strong downregulation of miR397–5p at 2 d in stamens and pistils was negatively correlated with changes in the expression of *SlLAC4* (Fig. 8, Additional file 2: Figure S4). However, no cleavage sites of miR397–5p were detected in *SlLAC4* transcripts in either stamens or pistils using RLM-5′-RACE. Therefore, the actual correlation between miR397–5p and laccase genes with respect to heat stress in tomato plants remains to be further characterized. However, considering that the lignin catabolic process was the most enriched GO term, we speculate that lignin synthesis or accumulation might be affected by high-temperature stress, resulting in changes in cell wall construction.

Overall, we found potential miRNAs that might participate in the regulation of stigma exsertion under high-temperature stress. Computational analyses combined with experimental approaches provided evidence that the miR398b-3p/*SlCSD1*, miR393-5p/*SlTIR1*, miR160a/*SlARF10/16*, miR156e-5p/*SlSPL15*, and miR397–5p/*LACs* cleavage cascades were tightly correlated with the regulation of the response to heat stress and metabolic pathways in stamens and pistils (Fig. 10). Previous studies have revealed that auxin and ROS both play important roles in regulating plant cell expansion and cell division through the regulation of cell-wall proteins and structures [73, 74]. Furthermore, ROS-mediated cell-wall loosening and extension growth are related with auxin [75]. Therefore, stigma exsertion under high-temperature treatment can be attributed to the differences in the metabolic pathway mediated by auxin and ROS signaling pathways in stamens and pistils. Certainly, this needs to be verified by further structural and functional studies.

## Conclusions

In this study, we attempted to illuminate the regulatory network of miRNAs participating in tomato stigma exsertion under high temperatures using integrated high-throughput sequencing and molecular biology approaches. A total of 69 and 30 heat-responsive miRNAs were identified from tomato stamen and pistil libraries, respectively. Comparisons and explorations of the expression of miRNAs and their targets in stamens and pistils upon heat stress provided evidence that some miRNA-target modules may play a critical role in the regulation of heat-stress responses and stigma exsertion. These results help elucidate the molecular mechanism of high-temperature-induced stigma exsertion in tomato plants.

## Methods

### Plant materials and treatments

The tomato cultivar “Micro-Tom” provided by the Tomato Genetic Resource Center, University of California, Davis, was grown in a growth chamber under standard conditions (25 ± 1 °C/20 ± 1 °C, 16 h day/8 h day/night photoperiod) with a relative humidity of 65–70%. When the first flower was fully opened, plants were exposed to 35 ± 1 °C/30 ± 1 °C (16 h day/ 8 h night) for 12 d, and untreated plants were kept at a control temperature of 25 ± 1 °C/20 ± 1 °C. A previous study showed that tomato flower bud size correlated with flower development stages [76]. Meanwhile, we previously found that the length of flower bud at the bicellular pollen stage was about 6.0–6.5 mm in the tomato cultivar ‘Micro-Tom’ [77]. To ensure a consistent flower-development period, stamens and pistils of flower buds of 6.0–6.5 mm length were separately sampled at 2 d and 12 d after heat stress (HS) treatment, resulting in the HS-2d and HS-12d libraries, respectively. Control samples were harvested at the same developmental stage from corresponding untreated plants at 2 d and 12 d, resulting in the CK-2d and CK-12d libraries, respectively. Each of the above treatments contained two biological replicates for sequencing. Hence, a total of 16 small RNA (sRNA) were constructed for stamens and pistils (Additional file 1: Table S1). The samples of each library were collected from 15 independent plants. Samples were immediately frozen in liquid nitrogen and stored at −70 °C until use. The length of at least ten stamens and pistils from ten independent plants were measured using vernier calipers with three biological replicates at different time points.

### Small RNA library construction and sequencing

The sRNAs were isolated using a mirVana miRNA isolation kit (Ambion, USA) according to the manufacturer’s instructions. Following purification, sRNAs of 18–30 nt were ligated with 5′- and 3′-RNA adaptors. Reverse transcription was then performed. Finally, the sRNA libraries were sequenced on an Illumina HiSeq 2500 system with the single-end 51-bp mode.

### Identification of known and novel miRNAs

Raw sRNA reads were processed to trim low-quality and adapter sequences. Trimmed sequences shorter than 15 nt were discarded. Then, rRNA, scRNA, snoRNA, snRNA, and tRNA were removed based on the Rfam [78] and NCBI GenBank databases (http://www.ncbi.nlm.nih.gov/GenBank/). Distinct sRNAs with ≥10 transcripts per million (TPM) in at least one sample and lengths of 18–30 nt were selected for miRNA identification. These sRNAs were aligned to the tomato genome sequences using Bowtie [79] with perfect match, and the flanking genome sequences (200 bp to each side) of sRNAs bearing no more than 20 unique genome hits were extracted and further folded with the RNAfold program [80]. The folded structures were checked with miRcheck to identify potential miRNA candidates [81, 82], which were further compared with miRBase [56] to screen for known miRNA candidates. Sequences with up to two mismatches were considered known miRNAs. The remaining miRNA candidates were further checked with the minimal free energies (MFEs) for their precursors and miRNA*s based on the parameters reported previously [83, 84]. Only miRNAs which met the criteria perfectly were considered novel miRNAs.

### Target prediction and GO and KEGG analyses

Putative miRNA targets were identified using psRNATarget [36] with default parameters. Gene Ontology (GO) annotation analysis for the predicted target genes was performed based on the GO database (http://www.geneontology.org/), whereas the KEGG database (http://www.genome.jp/kegg/) was utilized to annotate the miRNA target genes.

### Quantitative real-time RT-PCR (qRT-PCR)

The expression of miRNAs and the correlation between miRNAs and their targets were evaluated by quantitative real-time PCR (qRT-PCR). Total RNA was extracted from stamens and pistils of tomato plants exposed to heat stress and control temperature at the same sampling time points using the mirVana miRNA isolation kit (Ambion). First strand cDNA was synthesized using the Mir-X miRNA First-Strand Synthesis Kit (Clontech) according to the manufacturer’s protocol. All qRT-PCR reactions were performed in a Bio-Rad CFX96 (Bio-Rad) using SYBR® Green Realtime PCR Master Mix (Toyobo). In brief, 1 μl of cDNA template was added to 7.5 μl of qPCR Mix with 0.5 μl each primer, and ddH_2_O to a final volume of 15 μl. PCR conditions were set at 95 °C 30 s, followed by 39 cycles of 95 °C for 10 s and 55 °C for 30 s, and final elongation at 65 °C for 10 s. All reactions were performed in triplicate for each sample. *SnoU6* (Solyc12g056290.1.1) [85] and *SlUbi3* (Solyc01g056940.2.1) [86] were used as the internal controls for expression analysis of miRNAs and target genes, respectively. The fold-changes of miRNAs and target genes were calculated using the 2^-ΔΔCt^ method [87]. The primers used in this study are listed in Additional file 1: Table S10.

### RLM-5′ RACE analysis

The selected miRNA-target cleavage sites were validated using the GeneRacer kit (Invitrogen) through RNA ligase-mediated rapid amplification of 5′-cDNA ends (RLM-5’RACE). Briefly, mRNAs were isolated from 200 μg of total RNA using the PolyATtract® mRNA Isolation System IV (Promega). The 5′ RNA adaptor was ligated to the decapped mRNA using the T4 RNA ligase, followed by reverse transcription using the Superscript III RT Reaction kit (Invitrogen). The diluted reverse transcription product was used to amplify the desired target genes using 5′- GeneRacer and 3′- gene-specific primers (Additional file 1: Table S11). The amplified product was analyzed on a 1% agarose gel and cloned into pGEMT-easy (Promega). Ten independent colonies were subjected to sequence analysis to confirm the cleavage site.

### Statistical analysis

Statistical analysis was performed to compare the expression levels of the miRNAs between the different libraries. The log_2_ ratio was regarded as a threshold to detect the fold changes of miRNAs expression levels between high temperature-treated and control libraries. The differences between these libraries were tested using the chi-square test.

### Availability of supporting data

Supporting data are included as Additional file 1: Table S1-Table S11, Additional file 2: Figure S1-Figure S4.

## Additional files

Additional file 1: Table S1. Evaluation of the correction of the reduplicate biological samples. **Table S2.** Known miRNAs identified from stamen and pistil libraries. **Table S3.** Differentially expressed known and novel miRNAs between stamen and pistil CK-2d libraries. **Table S4.** Novel miRNAs identified from stamen and pistil libraries. **Table S5.** Differentially expressed miRNAs shared between stamen and pistil under heat stress condition. **Table S6.** Stamen specific differentially expressed miRNAs. **Table S7.** Pistil specific differentially expressed miRNAs. **Table S8.** Target genes of differentially expressed miRNAs in stamen. **Table S9.** Target genes of differentially expressed miRNAs in pistil. **Table S10.** List of primers used for qRT-PCR analysis. **Table S11.** List of primers used for RLM-5′ RACE analysis. (XLS 299 kb)

Additional file 2: Figure S1. Nucleotide bias at each position of identified miRNAs. Y-axis: frequency of A/U/G/C; X-axis: position in miRNAs. **Figure S2.** Real-time quantitative PCR validation of nine heat-responsive miRNAs in stamen (a) and pistil (b), respectively. Y-axis shows the log_2_ ratio of miRNAs expression in HS versus CK. *SnoU6* was used as the internal control. Each bar represents the mean ± SE of triplicated assays. **Figure S3.** Distribution of tRNAs, snoRNAs, and snRNAs in stamen and pistil libraries. Y-axis: frequency of each category of small RNAs. **Figure S4.** qRT-PCR analysis of the expression of *SlLAC4* in stamen (a) and pistil (b) under heat-stress treatment. *SlUbi3* was used as the internal control. Each bar represents the mean ± SE of triplicated assays. (ZIP 508 kb)

## Abbreviations

AGO: Argonaute
GO: Gene Ontology
HS: Heat stress
KEGG: Kyoto Encyclopedia of Genes and Genomes
MFE: Minimum free energy
qRT-PCR: Reverse transcription-quantitative polymerase chain reaction
RLM-5′ RACE: RNA ligase-mediated rapid amplification of 5′ cDNA ends
rRNA: Ribosomal RNA
snoRNA: Small nuclear RNA
snRNA: Small nuclear ribonucleic acids
TPM: Transcripts per million
tRNA: Transfer RNA
