# Supplementary figures and images for: Identification and expression profiling of microRNAs involved in the stigma exsertion under high-temperature stress in tomato
Source: BMC Genomics. 2017 Nov 2;18:843. doi: 10.1186/s12864-017-4238-9 (PMC5668977; doi:10.1186/s12864-017-4238-9)

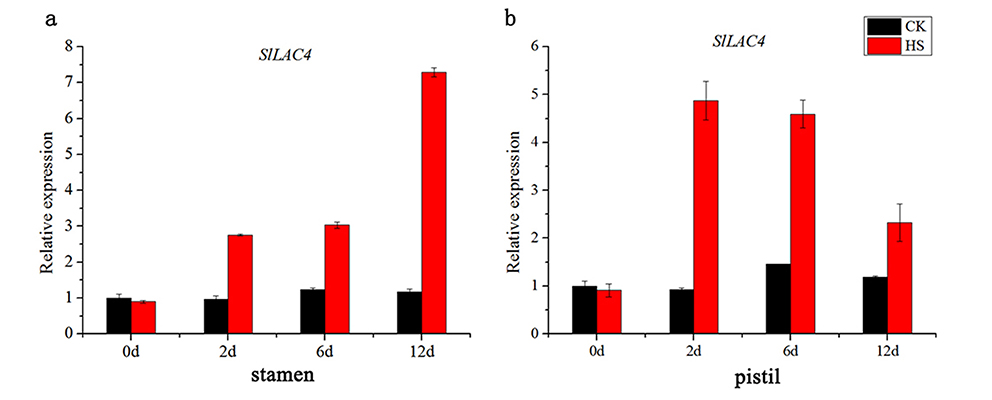

Supplement: Supplementary file 2 — Nucleotide bias at each position of identified miRNAs. Y-axis: frequency of A/U/G/C; X-axis: position in miRNAs. Figure S2. Real-time quantitative PCR validation of nine heat-responsive miRNAs in stamen (a) and pistil (b), respectively. Y-axis shows the log2 ratio of miRNAs expression in HS versus CK. SnoU6 was used as the internal control. Each bar represents the mean ± SE of triplicated assays. Figure S3. Distribution of tRNAs, snoRNAs, and snRNAs in stamen and pistil libraries. Y-axis: frequency of each category of small RNAs. Figure S4. qRT-PCR analysis of the expression of SlLAC4 in stamen (a) and pistil (b) under heat-stress treatment. SlUbi3 was used as the internal control. Each bar represents the mean ± SE of triplicated assays. (ZIP 508 kb) [file 12864_2017_4238_MOESM2_ESM.zip › Additional file 2 Figure S4.jpg]

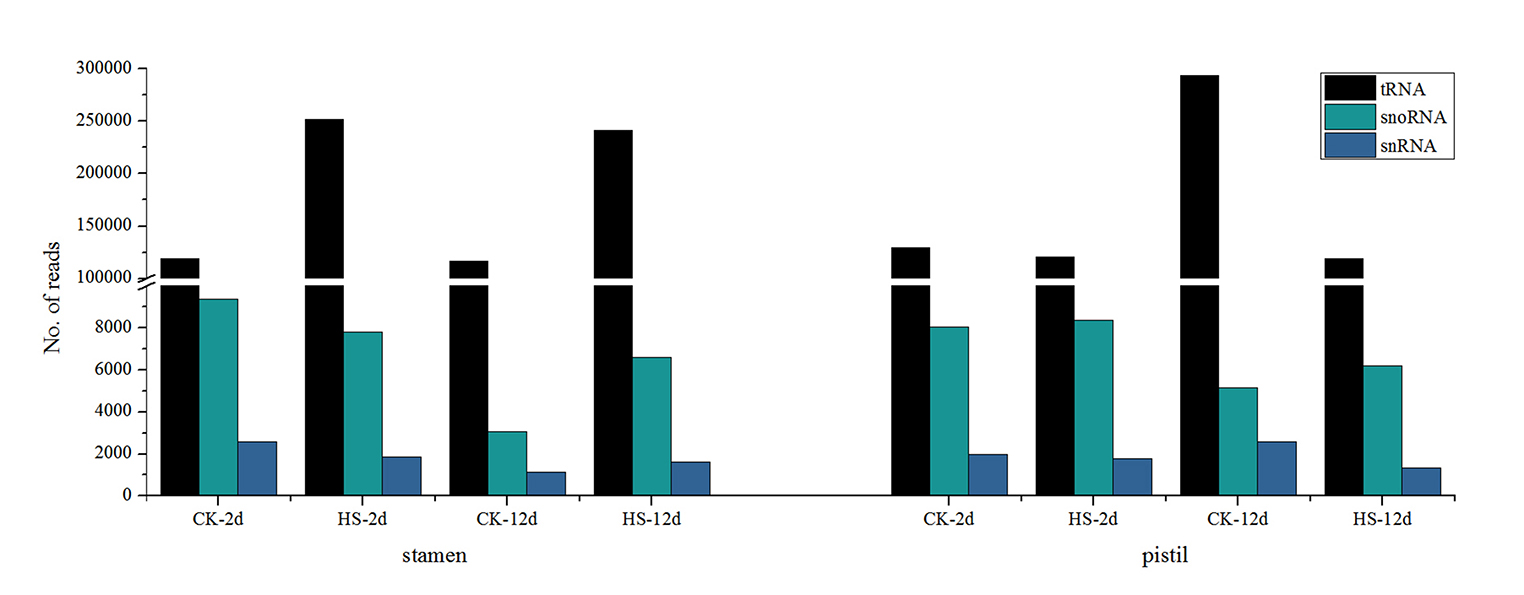

Supplement: Supplementary file 2 — Nucleotide bias at each position of identified miRNAs. Y-axis: frequency of A/U/G/C; X-axis: position in miRNAs. Figure S2. Real-time quantitative PCR validation of nine heat-responsive miRNAs in stamen (a) and pistil (b), respectively. Y-axis shows the log2 ratio of miRNAs expression in HS versus CK. SnoU6 was used as the internal control. Each bar represents the mean ± SE of triplicated assays. Figure S3. Distribution of tRNAs, snoRNAs, and snRNAs in stamen and pistil libraries. Y-axis: frequency of each category of small RNAs. Figure S4. qRT-PCR analysis of the expression of SlLAC4 in stamen (a) and pistil (b) under heat-stress treatment. SlUbi3 was used as the internal control. Each bar represents the mean ± SE of triplicated assays. (ZIP 508 kb) [file 12864_2017_4238_MOESM2_ESM.zip › Additional file 2 Figure S3.jpg]

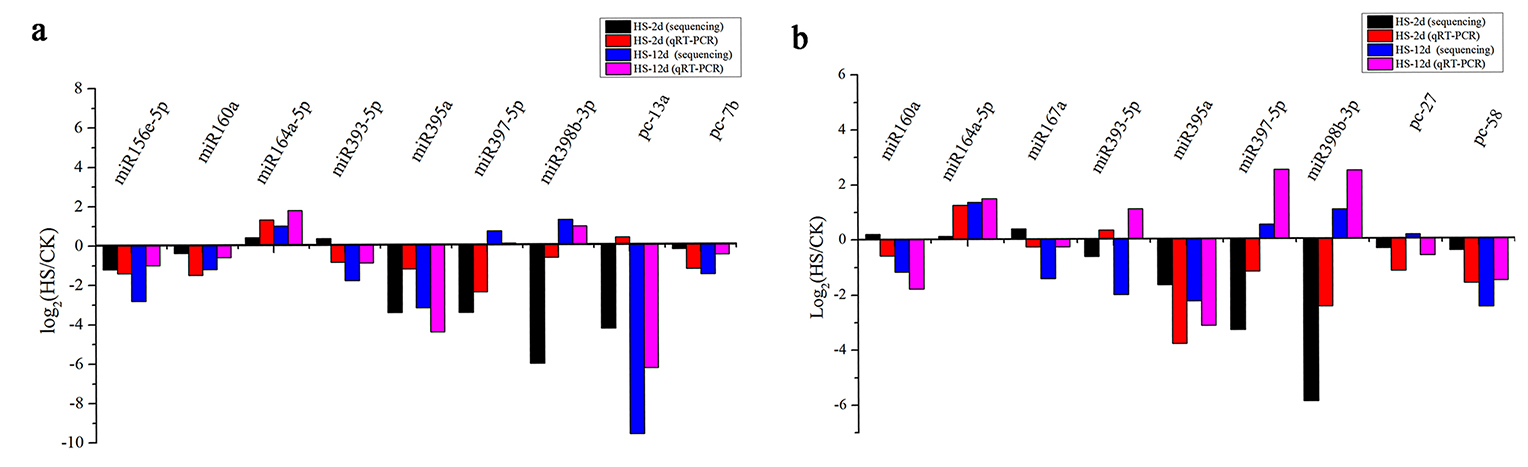

Supplement: Supplementary file 2 — Nucleotide bias at each position of identified miRNAs. Y-axis: frequency of A/U/G/C; X-axis: position in miRNAs. Figure S2. Real-time quantitative PCR validation of nine heat-responsive miRNAs in stamen (a) and pistil (b), respectively. Y-axis shows the log2 ratio of miRNAs expression in HS versus CK. SnoU6 was used as the internal control. Each bar represents the mean ± SE of triplicated assays. Figure S3. Distribution of tRNAs, snoRNAs, and snRNAs in stamen and pistil libraries. Y-axis: frequency of each category of small RNAs. Figure S4. qRT-PCR analysis of the expression of SlLAC4 in stamen (a) and pistil (b) under heat-stress treatment. SlUbi3 was used as the internal control. Each bar represents the mean ± SE of triplicated assays. (ZIP 508 kb) [file 12864_2017_4238_MOESM2_ESM.zip › Additional file 2 Figure S2.jpg]

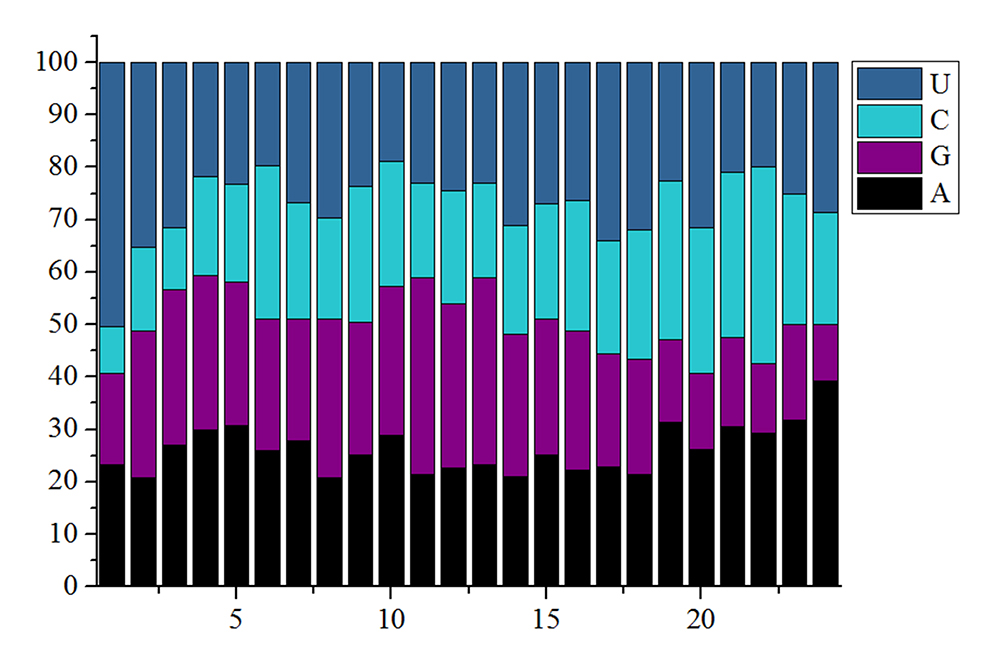

Supplement: Supplementary file 2 — Nucleotide bias at each position of identified miRNAs. Y-axis: frequency of A/U/G/C; X-axis: position in miRNAs. Figure S2. Real-time quantitative PCR validation of nine heat-responsive miRNAs in stamen (a) and pistil (b), respectively. Y-axis shows the log2 ratio of miRNAs expression in HS versus CK. SnoU6 was used as the internal control. Each bar represents the mean ± SE of triplicated assays. Figure S3. Distribution of tRNAs, snoRNAs, and snRNAs in stamen and pistil libraries. Y-axis: frequency of each category of small RNAs. Figure S4. qRT-PCR analysis of the expression of SlLAC4 in stamen (a) and pistil (b) under heat-stress treatment. SlUbi3 was used as the internal control. Each bar represents the mean ± SE of triplicated assays. (ZIP 508 kb) [file 12864_2017_4238_MOESM2_ESM.zip › Additional file 2 Figure S1.jpg]
